# Neurodevelopmental Tics with Co-Morbid Functional Tic-like Behaviors: Diagnostic Challenges of a Complex Tourette Syndrome Phenotype

**DOI:** 10.3390/brainsci15050435

**Published:** 2025-04-23

**Authors:** Andrea Eugenio Cavanna, Virginia Caimi, Elisa Capriolo, Matteo Marinoni, Gabriele Arienti, Anna Riva, Renata Nacinovich, Stefano Seri

**Affiliations:** 1 Department of Neuropsychiatry, National Centre for Mental Health, Birmingham and Solihull Mental Health NHS Foundation Trust, Birmingham B15 2FG, UK; 2 School of Medical Sciences, College of Medicine and Health, University of Birmingham, Birmingham B15 2TT, UK; 3 College of Health and Life Sciences, Institute of Health and Neurodevelopment, Aston University, Birmingham B4 7ET, UK; s.seri@aston.ac.uk; 4 Sobell Department of Motor Neuroscience and Movement Disorders, Institute of Neurology, University College London, London WC1E 6BT, UK; 5 Department of Child Neuropsychiatry, Fondazione IRCCS San Gerardo dei Tintori, 20900 Monza, Italy; v.caimi@campus.unimib.it (V.C.); gabriele.arienti@irccs-sangerardo.it (G.A.);; 6 School of Medicine and Surgery, University of Milano-Bicocca, 20125 Milan, Italy

**Keywords:** neurodevelopmental tics, Tourette syndrome, functional tic-like behaviors, functional overlay

## Abstract

**Background/Objectives**: The co-morbidity between neurodevelopmental tics and functional tic-like behaviors (FTBs) in patients with Tourette syndrome (TS) is relatively under-investigated. The demographic and clinical characteristics of a large sample of patients with TS who presented with co-morbid FTBs (functional overlay) were assessed to raise awareness of this complex clinical presentation and to shed light on the differential diagnosis between the two conditions. **Methods**: We analyzed the clinical data of 63 patients (44 females, mean age 24 years, range 13–40) with pre-existing TS who (sub)acutely developed co-morbid FTBs (TS + FTBs) after the onset of the COVID-19 pandemic and compared them with 63 age- and gender-matched controls with TS (neurodevelopmental tics only). The diagnosis of co-morbid FTBs was validated by the European Society for the Study of Tourette Syndrome (ESSTS) criteria. **Results**: Complex vocal tics (*p* < 0.001), including coprolalia (*p* = 0.002), and self-injurious behaviors (*p* < 0.001), often as part of tic attacks (*p* < 0.001), were confirmed to be more commonly reported by the group of patients with TS + FTBs, who were also more likely to present with anxiety (*p* < 0.001) and other functional neurological symptoms (*p* < 0.001) compared to patients with TS. **Conclusions**: Patients with TS and co-morbid FTBs can pose significant diagnostic and treatment challenges. By systematically applying ESSTS criteria, we confirmed specific red flags for the diagnosis of functional overlay in patients with TS. The correct identification of this composite clinical phenotype plays a key role in preventing the misdiagnosis of treatment-resistant TS and implementing tailored treatment interventions.

## 1. Introduction

Tourette syndrome (TS) is listed in the DSM-5 as a neurodevelopmental disorder characterized by multiple motor and vocal tics with an average age at onset of 6 years and an estimated prevalence of 0.3–1% in school-aged children [1,2]. Neurodevelopmental tics are three-to-four times more common in males than females [3]. The clinical phenomenology is mainly characterized by simple motor tics (e.g., eye blinking, facial grimacing, shoulder shrugging) and simple vocal tics (e.g., throat clearing, grunting, sniffing) which tend to develop according to a rostrocaudal distribution [4,5,6]. More complex manifestations such as coprolalia are reported by up to 30% of patients, with similar prevalence rates for non-obscene socially inappropriate behaviors [7,8,9]. Up to 90% of patients present with psychiatric comorbidities, most commonly tic-related obsessive-compulsive disorder (OCD) and attention-deficit/hyperactivity disorder (ADHD), that bear a significant impact on their health-related quality of life, often leading to academic difficulties, social isolation, and an increased risk of depression and anxiety [10,11,12,13,14].

Functional tic-like behaviors (FTBs) are a subset of functional movement disorders, characterized by sudden-onset motor and vocal manifestations that resemble neurodevelopmental tics [15,16]. Historically considered to be rare, FTBs were the presenting symptoms of an outbreak during the COVID-19 pandemic (referred to as a “pandemic within a pandemic”), particularly in female adolescents and young adults [17,18,19,20,21,22]. This phenomenon has been attributed at least in part to psychosocial stressors related to the pandemic and increased exposure to tic-related content on social media platforms [23,24,25,26]. It has been shown that FTBs present distinct clinical features that differentiate them from neurodevelopmental tics [16]. Key diagnostic clues include a later age of onset (typically adolescence or early adulthood), an abrupt onset and rapid progression (not following a rostrocaudal distribution), greater prevalence of complex tics—including coprophenomena, self-injurious behaviors and tic attacks—over simple ones, and symptoms that worsen in social settings [16,27,28]. While patients with neurodevelopmental tics often describe subjective premonitory urges and tic suppressibility, individuals with FTBs frequently report an inability to suppress their symptoms [29,30]. Additionally, family history of neurodevelopmental tics is common in TS but absent in most cases of FTBs [31].

Neurodevelopmental tics and FTBs are characterized by different etiological and pathophysiological mechanisms. The heritability pattern of TS is characterized by genetic heterogeneity, with *HDC* and *SLITRK1* being the only two genes currently associated with TS in the OMIM database (https://omim.org/entry/137580, accessed on 17 April 2025). Moreover, dysfunction of dopaminergic pathways within the cortico-striato-thalamo-cortical circuits has been reported in patients with TS but is not thought to be part of the mechanistic processes underlying FTBs [16,32]. Accordingly, pharmacological treatments for neurodevelopmental tics, such as antidopaminergic agents and alpha-adrenergic agonists, have shown little to no efficacy in FTBs [1,33,34]. Psychoeducation, cognitive behavioral therapy, and stress management strategies are the current mainstays of treatment approaches for FTBs [16]. It has been suggested that over 70% of patients with FTBs improve within six to twelve months, particularly when underlying anxiety and affective symptoms are adequately managed [35]. However, some individuals experience persistent functional symptoms even after tic resolution [36,37].

While our understanding of FTBs has increased considerably since the onset of the COVID-19 pandemic, there is still limited understanding of the co-occurrence of neurodevelopmental tics and FTBs in individuals with a pre-existing diagnosis of TS. In the present study, we set out to examine the demographic and clinical features of a large group of patients with TS who presented with co-morbid FTBs (functional overlay), aiming to increase awareness of this complex clinical presentation and assist clinicians in the differential diagnosis between the two conditions presenting in the same individual.

## 2. Materials and Methods

We identified a sample of patients with Tourette syndrome (neurodevelopmental tics) and functional overlay (co-morbid FTBs) by retrospectively cross-checking two large clinical datasets from the specialist Tourette Syndrome Clinic, Department of Neuropsychiatry, National Centre for Mental Health, Birmingham, United Kingdom: (1) a clinical sample of 726 consecutive patients with TS assessed between July 2008 and February 2025 and (2) a clinical sample of 185 consecutive patients who presented with tic-like behaviors and received a clinically definite diagnosis of functional tics after the publication of the European Society for the Study of Tourette Syndrome (ESSTS) criteria for the clinical diagnosis of FTBs (January 2023–March 2025).

The striking increase in FTBs since the onset of the COVID-19 pandemic prompted members of the ESSTS to develop a set of diagnostic criteria to support the diagnosis of this functional neurological phenotype [38]. The following three major criteria were proposed: (1) age of onset of 12 years or older, (2) rapid evolution of symptoms, and (3) presence of four out of nine specific phenomenological features: multiple types of tic-like behaviors, with a higher frequency of complex tic-like behaviors than simple ones (3a); inconsistent tic-like behaviors that are not repetitive or stereotyped (3b); complex motor tic-like behaviors including context-dependent or violent/offensive tic-like behaviors (3c); evolution of tic-like behaviors not following the rostrocaudal progression (3d); coprolalia (3e); tic-like behaviors likely to be influenced by popular culture or social interactions (3f); frequent fluctuations in intensity and frequency throughout the day (3g); new tic-like behaviors emerging regularly (3h). The same group of experts also proposed two minor criteria: (1) co-morbidity with anxiety and depression and (2) presence of other functional neurological symptoms. According to the authors, a clinically definite diagnosis of FTBs can be confirmed by the presence of all three major criteria, whereas a clinically probable diagnosis of FTBs can be confirmed by the presence of two major criteria and one minor criterion. The specificity of the phenomenological criteria for FTBs was recently tested in a sample of 156 patients with primary tic disorders (of whom 132 were diagnosed with TS), supporting the use of the ESSTS criteria in clinical practice [39].

Each patient was assessed by a behavioral neurologist with over 20 years of clinical experience with both primary tic disorders and functional neurological disorders (AEC). Comprehensive demographic and clinical data were systematically collected in order to confirm the diagnosis of either TS (neurodevelopmental tics) or functional neurological disorder (functional tics) according to DSM-5 criteria [2]. The assessment was based on the National Hospital Interview Schedule for Tourette syndrome [40], a detailed semi-structured interview schedule originally validated in patients with neurodevelopmental tics and adapted for use in patients with functional tics by including key items relevant to functional movement disorders [41]. Demographic and clinical data included gender, age at assessment, age and type of onset, environmental/psychological triggers and clinical phenomenology of tics, family history of tic disorder, psychiatric co-morbidities, and treatment interventions. For the purpose of the present study, we systematically screened the medical records of all patients who received DSM-validated diagnoses of both (1) Tourette syndrome and (2) functional neurological disorder (conversion disorder)—motor subtype presenting with functional tics. We included patients of all ages, whereas we excluded patients with incomplete or missing data.

All patients provided informed consent to participate in the study, which was approved by the local section of the National Research Ethics Service. Anonymized data were stored on Microsoft Excel 2021. The Statistical Package for the Social Sciences for Windows (SPSS Inc., Chicago, IL, USA, version 25) was used to perform all statistical analyses. This retrospective study was conducted using descriptive statistics to illustrate the demographic and clinical characteristics of the patients with FTBs. We used Fisher’s exact test for dichotomous variables and Student’s t-test (independent-samples, un-paired two-tailed comparison) for continuous variables to assess possible differences between the group of patients with TS + FTBs and the matched control group with TS. The Bonferroni correction for multiple comparisons was systematically applied. Finally, we assessed possible correlations between patient age and the severity of TS using Pearson’s correlation coefficient.

## 3. Results

We retrieved data from 63 patients with a longstanding diagnosis of TS who received a clinically definite diagnosis of co-morbid FTBs (functional overlay) validated by ESSTS criteria (Table 1). The four phenomenological criteria that most often contributed to the diagnosis of FTBs in patients with TS + FTBs were evolution of tic-like behaviors not following the rostrocaudal progression that is typical of neurodevelopmental tics (93.7%); presence of complex motor tic-like behaviors including context-dependent or violent/offensive tics such as throwing, hitting, and tic-related self-injurious behaviors (69.8%); functional coprolalia (63.5%); and frequent fluctuations in intensity and frequency throughout the day (60.3%).

At the time of their specialist assessment, the mean age of the patients with TS + FTBs was 24.0 ± 9.7 years (range: 13–40 years), and the majority of them were females (N = 44, 69.8%). Their clinical characteristics are shown in Table 2, alongside a comparison with an independent sample of age- and sex-matched controls with TS who did not develop co-morbid FTBs. There was no significant difference in neurodevelopmental tic severity between patients with TS + FTBs and patients with TS only (*p* = 0.532). There was no significant correlation between patient age and the severity of TS in either the TS + FTBs group (R = 0.029, *p* = 0.822) or the TS only group (R = 0.026, *p* = 0.840).

All patients developed FTBs after the onset of their neurodevelopmental tics. Specifically, the mean age at neurodevelopmental tic onset was 7.1 ± 3.3 years (range 2–15 years), whereas the mean age at FTBs onset was 22.4 ± 9.4 years (range: 12–60 years), on average 15.3 years later (*p* < 0.001). The onset of FTBs was reported as a sudden worsening of the clinical presentation after a longstanding and relatively stable course of the disease. After the diagnosis of co-morbid FTBs had been established, N = 38 patients (60.3%) were subjectively able to differentiate their functional overlay from their pre-existing neurodevelopmental tic disorder, based on the absence of their usual premonitory urges.

With regard to clinical phenomenology, patients with TS + FTBs were significantly more likely to present with complex vocalizations (*p* < 0.001), including functional coprolalia (*p* = 0.002), than patients with TS. Their motor manifestations lacked the rostrocaudal distribution that characterizes neurodevelopmental tics (*p* < 0.001) and were significantly more likely to involve complex repetitive movements of the limbs, including self-hitting and other forms of repetitive self-injurious behaviors (*p* < 0.001). Clusters of FTBs or tic attacks were reported by 23/63 patients with functional overlay (36.5%) versus none of the matched patients with TS (*p* < 0.001). The two patient groups differed significantly in their co-morbidity profiles, as patients with TS + FTBs reported higher rates of other neurodevelopmental conditions (high functioning autism spectrum disorder, ASD: *p* < 0.001; ADHD: *p* = 0.001) and anxiety disorders (*p* < 0.001), as well as other functional neurological disorders (*p* < 0.001). Specifically, N = 19 (30.2%) patients with TS + FTBs reported non-epileptic attacks, N = 8 (12.7%) functional dystonia, N = 3 (4.8%) functional tremor, and N = 3 (4.8%) functional weakness. In terms of treatment interventions, both patient groups had similar proportions of patients taking pharmacotherapy (TS + FTBs: 65.1%; TS: 58.7%) and receiving psychotherapy interventions (TS + FTBs: 31.7%; TS: 25.4%). Pharmacotherapy of patients with TS and functional overlay was as follows: N = 30 patients (47.6%) were prescribed serotonergic medications (N = 12 Sertraline, N = 8 Citalopram, N = 4 Fluoxetine, N = 3 Escitalopram, N = 2 Amitriptyline, N = 1 Fluvoxamine), N = 19 patients (30.2%) antidopaminergic agents (N = 12 Aripiprazole, N = 6 Risperidone, N = 1 Haloperidol), N = 13 (20.6%) alpha-2 agonists (N = 11 Clonidine, N = 2 Guanfacine), N = 6 (9.5%) benzodiazepines (N = 5 Diazepam, N = 1 Lorazepam), N = 5 (7.9%) beta-blockers (N = 4 Propranolol, N = 1 Bisoprolol), and N = 11 (17.5%) other pharmacological options (N = 2 Duloxetine, N = 2 Mirtazapine, N = 2 Promethazine, N = 2 Topiramate, N = 1 Atomoxetine, N = 1 Melatonin, N = 1 Venlafaxine).

## 4. Discussion

**Figure 1:**
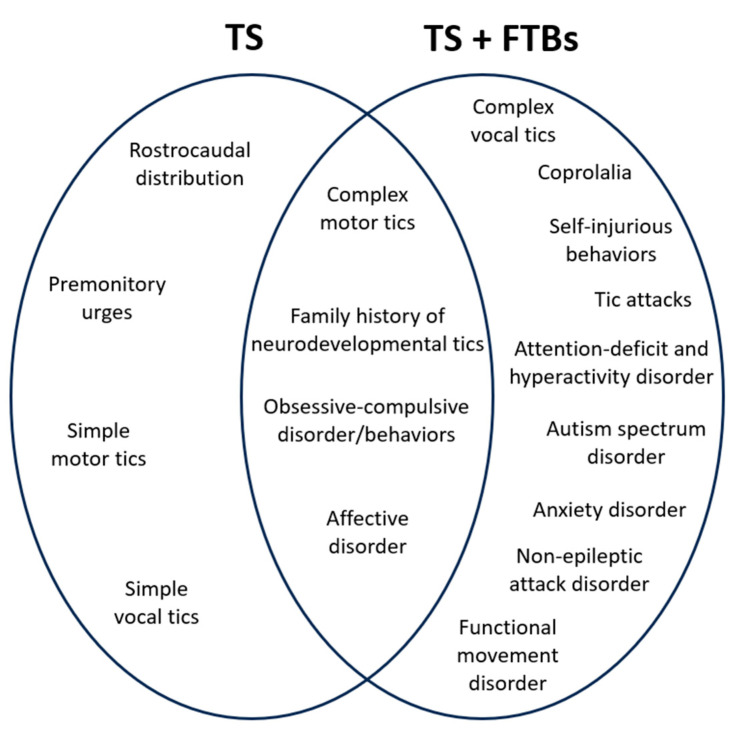
Clinical characteristics of Tourette syndrome (TS) with and without functional tic-like behaviors (FTBs).

To the best of our knowledge, the present study provides original data on the second largest cohort of patients with TS and co-morbid FTBs. This is also the first study to implement the ESSTS criteria for the diagnosis of functional overlay in this TS phenotype. Our sample consisted of adolescent and young adults, with a striking preponderance of female gender, who (sub)acutely developed new manifestations not following the typical rostrocaudal distribution of their longstanding neurodevelopmental tics. Neurodevelopmental tic severity was comparable to that of matched TS controls. Subjectively, FTBs appeared to be distinguishable from neurodevelopmental tics based on the absence of their usual premonitory urges. Complex vocal tics (especially functional coprolalia), tic-related self-injurious behaviors, and tic attacks were confirmed to be more commonly reported by the group of patients with TS + FTBs, who were also more likely to present with anxiety and other neurodevelopmental and functional neurological disorders compared to patients with TS. Increased rates of anxiety have been reported both in patients with TS [1] and in patients with functional movement disorders [41]. Of particular note is the clinically relevant overlap in the OCD behavioral profile between the TS + FTBs group and the TS only group, suggesting an intrinsic co-morbidity pattern between neurodevelopmental tics and tic-related obsessive-compulsive behaviors [11], which does not seem to be affected by the presence of FTBs. Conversely, we found that other neurodevelopmental conditions such as ADHD and ASD, which are known to be associated with TS [1], were reported with significantly higher prevalence in the TS + FTBs group, suggesting that the development of a functional overlay might be facilitated by pre-existing (high functioning) ASD and ADHD. The absence of differences in family history of neurodevelopmental tics between patients with TS + FTBs and patients with TS who did not develop FTBs is likely to reflect the shared heritability of TS, with no additional effect related to co-morbid FTBs. The full list of overlapping clinical characteristics between the two patient groups is shown in Figure 1.

In the pre-pandemic era, reports of patients with TS who subsequently developed co-morbid FTBs were extremely rare [42]. In 1992, Kurlan et al. reported the case of an 18-year-old female patient with mild TS who had developed complex movements at the age of 16, including slumping in a chair or onto the floor with rhythmic tonic-clonic movements of all limbs [43]. Two years later, Dooley et al. reported two similar cases [44]. The first one was a 9-year-old girl who developed unusual movements, such as situation- and context-specific jumping on a chair, followed by thrashing movements of the arms, legs, and trunk, two years after the onset of her neurodevelopmental tics. The second one was a 16-year-old girl who began experiencing repetitive complex movements described as “wild thrashing motions of all four limbs and her trunk” six years after the onset of her neurodevelopmental tics. In the latter case, FTBs resolved after a history of sexual abuse emerged and was addressed during hospitalization. In 2014, Janik et al. published a study where they found a prevalence rate of 1.9% for FTBs (N = 5, ages 17–51, with one female) among a cohort of 268 patients with neurodevelopmental tic disorders [45]. In all cases, FTBs developed after the onset of neurodevelopmental tics and were characterized by complex movements and absence of the usual premonitory urges.

During the COVID-19 pandemic, Kurvits et al. reported three cases of aggression toward others misdiagnosed as neurodevelopmental tics [46]. One of these subjects was a 20-year-old female who developed coprolalia and aggressive behaviors, including throwing objects and pushing others, which were not part of her longstanding TS diagnosis (simple repetitive movements consistent with childhood tics). Of note, she was also diagnosed with ADHD and reported non-epileptic attacks alongside her FTBs. Fremer et al. reported data on 32 patients attending a specialist TS clinic in Germany who received a diagnosis of functional tics after exposure to relevant social media content between May 2019 and September 2021 [47]. Of these, 15 patients were identified as having a diagnosis of TS with functional overlay (functional tics). The authors compared the characteristics of tics between their 15 patients with functional tics plus co-morbid TS (“functional tics plus”) and 17 patients with functional tics in the absence of TS (“functional tics only”). Patients in the “functional tics only” group reported significantly higher rates of abrupt onset of symptoms and lower rates of co-morbid obsessive-compulsive behaviors, compared to the “functional tics plus” group. In 2022, our group reported a case series of 10 patients with TS (9 females, ages 13–24) who had developed a functional overlay consisting of FTBs on average 9 years after the onset of their neurodevelopmental tics [48]. A within-subject comparison between neurodevelopmental tics and functional tics revealed that the latter ones were significantly more likely to be associated with a (sub)acute onset in the absence of a rostrocaudal distribution. The higher prevalence of complex manifestations, including tic-related self-injurious behaviors, coprolalia, and non-obscene socially inappropriate behaviors in the context of FTBs did not reach statistical significance in this relatively small sample.

In 2023, Müller-Vahl et al. published data on the largest sample of patients with TS and co-morbid FTBs to date (N = 71; 38.0% females, mean age 21.5 years, range 11–55) [49]. Patients in this clinical sample developed FTBs on average 15 years after the onset of their neurodevelopmental tics. As in our sample, the onset of FTBs was abrupt and did not follow the rostrocaudal distribution that is typical of neurodevelopmental tics. The clinical phenomenology was broadly in line with our findings, as it was characterized by complex manifestations, including throwing and hitting movements, self-injurious behaviors, coprolalia, and tic attacks. Specifically, 55% of patients presented with self-hitting and other self-injurious behaviors, 46% with tic attacks, and 38% with variable functional obscene words. These findings replicate the results of phenomenological studies conducted in cohorts of patients with FTBs only [27]. The spectrum of psychiatric comorbidities overlapped only partially with our results: Müller-Vahl et al. [49] found an increased rate of OCD in patients with TS + FTBs, whereas our sample was characterized by comorbidity with other neurodevelopmental conditions (high functioning ASD and ADHD), possibly reflecting geographical variations in diagnostic thresholds [50,51]. Both psychological and environmental stressors were identified as significant contributors to the development of the functional overlay. Specifically, exposure to tic-related content on social media platforms (“mass social media-induced illness”) has been proposed as a key factor in the surge of FTBs by the German group [49,52]. Mass social-media-induced illness is a digital-age variant of mass psychogenic illness [47]. Notably, it has been shown that the phenomenology of tics portrayed on social media differs significantly from genuine TS, featuring exaggerated, environmentally triggered, and often self-injurious behaviors [53,54]. According to this model, exposure to the contents produced by a popular male influencer based in Germany might have contributed to the relatively higher prevalence of male gender in the sample described by Müller-Vahl et al. compared to both the present study on TS + FTBs and other studies on FTBs outside Germany [16,27,49].

This study has several limitations. While we recruited the second-largest sample of patients with TS who developed FTBs since the onset of the COVID-19 pandemic, the sample size remains relatively small. Additionally, referral bias must be considered, as participants were recruited from a specialist clinic and may not be representative of the broader population of patients with TS and FTBs. Another limitation is inherent to the retrospective design of the study, which limits causal inferences. Although the standardized assessment procedure reduced risks of bias from incomplete documentation, the lack of longitudinal or interventional data weakens recommendations for clinical management. Finally, despite the use of ESSTS criteria for diagnostic validation purposes, the clinical approach to FTBs might involve circular reasoning due to a lack of clinical benchmarks [55]. Clinical features like inconsistency and incongruity, which are commonly used to assist the diagnosis of other functional movement disorders, prove challenging when applied to patients with tics.

In summary, our data suggest that FTBs are likely to be a relatively common co-morbidity in patients with TS, similarly to the well-established concept of functional overlay in other hyperkinetic movement disorders [56] and epilepsy [57]. Abrupt onset in adolescence or early adulthood, a higher-than-expected proportion of females, complex vocalizations including coprolalia, tic-related self-injurious behaviors, tic attacks, co-morbid anxiety and other functional neurological disorders were confirmed to be red flags indicating the (sub)acute development of a functional overlay in patients with a longstanding history of TS. Evidence that co-morbid FTBs as part of a functional overlay in patients with TS are likely to be more prevalent than previously recognized has implications in terms of treatment interventions. Müller-Vahl et al. highlighted the risk of iatrogenic harm in patients with TS + FTBs who could be mistakenly classified as having treatment-resistant TS and referred to more invasive treatment interventions such as deep brain stimulation [49].

## 5. Conclusions

It has been suggested that about one third of patients with TS present with co-morbid movement disorders that should be differentiated and distinguished from neurodevelopmental tics, as their etiopathogenesis and treatment are likely to be different [42]. Our study addressed a clinically significant and timely issue: the co-occurrence of neurodevelopmental tics (TS) and FTBs, particularly since the onset of the COVID-19 pandemic. The surge in FTBs during the pandemic and their overlap with pre-existing TS pose diagnostic and therapeutic challenges, of interest to both clinicians and researchers across neurology and psychiatry. The results from the second largest cohort of patients with TS and co-morbid FTBs highlight key differences between TS + FTBs and TS-only patients, including higher rates of complex vocal tics, self-injurious behaviors, anxiety, and functional neurological symptoms in the TS + FTBs group. These findings are clinically meaningful as they identify indicators (e.g., abrupt onset, lack of rostrocaudal progression) that aid in distinguishing FTBs from pre-existing neurodevelopmental tics. Finally, the replication of prior findings on the phenomenology of FTBs in a TS population strengthens the validity of the ESSTS criteria. Finally, our findings prompt treating clinicians to develop and implement tailored interventions to improve health-related quality of life in patients with this complex clinical phenotype.

## Figures and Tables

**Table 1:** European Society for the Study of Tourette Syndrome (ESSTS) criteria supporting the clinical diagnosis of functional tic-like behaviors in patients with a previous diagnosis of TS (N = 63).

ESSTS Criteria	N (%)
Major criterion 1 (age of onset ≥12)	63 (100%)
Major criterion 2 (rapid evolution of symptoms)	63 (100%)
Major criterion 3a (multiple types of tic-like behaviors, with a higher frequency of complex than simple ones)	36 (57.1%)
Major criterion 3b (inconsistent tic-like behaviors that are not repetitive or stereotyped)	21 (33.3%)
Major criterion 3c (complex motor tic-like behaviors including context-dependent or violent/offensive tics)	44 (69.8%)
Major criterion 3d (evolution of tic-like behaviors not following the rostrocaudal progression)	59 (93.7%)
Major criterion 3e (coprolalia)	40 (63.5%)
Major criterion 3f (tic-like behaviors likely to be influenced by popular culture or social interactions)	26 (41.3%)
Major criterion 3g (frequent fluctuations in intensity and frequency throughout the day)	38 (60.3%)
Major criterion 3h (new tic-like behaviors emerging regularly)	29 (46.0%)
Minor criterion 1 (comorbidity with anxiety/depression)	49 (77.8%)
Minor criterion 2 (presence of other functional neurological symptoms)	24 (38.1%)

**Table 2:** Clinical characteristics of patients with Tourette syndrome (TS) and co-morbid functional tic-like behaviors (FTLBs) compared to an independent sample of age- and sex-matched controls with TS (N = 63).

	**TS + FTLBs**	**TS**	***p*-Value**
Age at onset—years (mean, sd, range) *	22.4 (±9.4) (12–60)	7.6 (±3.8) (1–17)	<0.001
Yale Global Tic Severity Scale tic severity score (mean, sd, range)	27.1 (±9.4) (7–45)	28.2 (±9.7) (9–49)	0.532
Rostrocaudal distribution (N, %) *	4 (6.3%)	49 (77.8%)	<0.001
Premonitory urges (N, %) *	25 (39.7%)	61 (96.8%)	<0.001
Simple motor tics (N, %) *	40 (63.5%)	63 (100%)	<0.001
Complex motor tics (N, %) *	57 (90.5%)	50 (79.4%)	0.134
Simple vocal tics (N, %) *	34 (54.0%)	63 (100%)	<0.001
Complex vocal tics (N, %) *	50 (79.4%)	26 (41.3%)	<0.001
Coprolalia (N, %) *	40 (63.5%)	22 (34.9%)	0.002
Tic-related self-injurious behaviors (N, %) *	38 (60.3%)	7 (11.1%)	<0.001
Tic attacks (N, %) *	23 (36.5%)	0 (0%)	<0.001
Family history of neurodevelopmental tics (N, %)	32 (50.8%)	31 (49.2%)	1
Obsessive-compulsive disorder (N, %)	13 (20.6%)	14 (22.2%)	1
Obsessive-compulsive behaviors (N, %)	46 (73.0%)	40 (63.5%)	0.214
Attention-deficit and hyperactivity disorder (N, %)	29 (46.0%)	11 (17.5%)	0.001
Autism spectrum disorder (N, %)	19 (30.2%)	3 (4.8%)	<0.001
Affective disorder (N, %)	33 (52.4%)	22 (34.9%)	0.072
Anxiety disorder (N, %)	45 (71.4%)	9 (14.3%)	<0.001
Non-epileptic attack disorder (N, %)	19 (30.2%)	0 (0%)	<0.001
Functional movement disorder (N, %) **	14 (22.2%)	0 (0%)	<0.001
Pharmacotherapy (N, %)	41 (65.1%)	37 (58.7%)	0.582
Psychotherapy (N, %)	20 (31.7%)	16 (25.4%)	0.555

* FTLBs in the group of patients with TS + FTLBs. ** Different from FTLBs.

## Data Availability

The data presented in this study are available on request from the corresponding author due to confidentiality issues.
